# Author Correction: Broadband slow-wave modulation in posterior and anterior cortex tracks distinct states of propofol-induced unconsciousness

**DOI:** 10.1038/s41598-024-68301-1

**Published:** 2024-08-15

**Authors:** Emily P. Stephen, Gladia C. Hotan, Eric T. Pierce, P. Grace Harrell, John L. Walsh, Emery N. Brown, Patrick L. Purdon

**Affiliations:** 1 Picower Institute for Learning and Memory, Massachusetts Institute of Technology, Cambridge, 02139 USA; 2 Department of Brain and Cognitive Sciences, Massachusetts Institute of Technology, Cambridge, 02139 USA; 3 Institute of Medical Engineering and Science, Massachusetts Institute of Technology, Cambridge, 02139 USA; 4 Department of Anesthesia, Critical Care and Pain Medicine, Massachusetts General Hospital, Boston, 02114 USA; 5 MIT-Harvard Health Sciences and Technology, Cambridge, 02142 USA; 6 Department of Anaesthesia, Harvard Medical School, Boston, 02115 USA

Correction to: *Scientific Reports* 10.1038/s41598-020-68756-y, published online 13 August 2020

The Competing interests section in the original version of this Article was incorrect.

“G.C.H., E.T.P., P.G.H., J.L.W., and E.N.B. declare no potential conflict of interest.”

now reads:

“E.N.B holds patents on anesthetic state monitoring and control. E.N.B. holds founding interest in PASCALL, a start-up developing physiological monitoring systems; receives royalties from intellectual property through Massachusetts General Hospital licensed to Masimo. The interests of E.N.B. were reviewed and are managed by Massachusetts General Hospital and Mass General Brigham in accordance with their conflict of interest policies.”

The original Article has been corrected.

